# Educational strategy for the development of skills in exchange transfusion: a randomized clinical trial protocol

**DOI:** 10.1186/s13063-020-04312-3

**Published:** 2020-05-07

**Authors:** María José Maldonado, Sergio Iván Agudelo, Juan David Suarez, Oscar Gamboa

**Affiliations:** 1 School of Medicine, Master in Medical Education, Universidad de La Sabana (University of La Sabana), Chía, Colombia; 2 School of Medicine, Universidad de La Sabana, Chía, Colombia

**Keywords:** Newborn, Simulation training, Virtual, Telemedicine, Objective Structured Clinical Examination (OSCE), Individual assessment, Safety training, Simulation centre

## Abstract

**Background:**

Exchange transfusion is a highly complex procedure that requires high levels of expertise. Trainee paediatricians do not have adequate training in exchange transfusion because opportunities to perform this procedure in practice are scarce. This protocol seeks to compare two educational interventions for exchange transfusion that allow students to develop competencies to perform the technique in an appropriate and safe way.

**Methods/design:**

This is a randomized parallel single-blind clinical trial with allocation by simple randomization to the educational intervention (simulation or a digital didactic environment). Students from the paediatric specialization who volunteer to participate will be included. A practical evaluation of the procedure will be performed through a simulated scenario using a standardized clinical case. The main outcome is defined as the result of evaluation using the Objective Structured Clinical Examination; superior performance will be defined when the percentage is greater than or equal to 85%, and non-superior performance will be defined when the result is less than 84%. The chi-square independence test or the Fisher exact test will be used to evaluate the effect of the interventions. Multivariate analysis will be performed using a non-conditional logistic regression model. Stata 15® software will be used.

**Discussion:**

Exchange transfusion is a procedure that requires expertise to achieve adequate outcomes. The inclusion of new educational strategies, such as simulation and digital didactic environments, is seen as a training option that can improve performance in clinical skills, reduce adverse events and increase the level of trust.

**Trial registration:**

ClinicalTrials.gov: NCT04070066. Registered on 28 August 2019. https://clinicaltrials.gov.

## Background

### Scientific background and justification

Exchange transfusion is a highly complex procedure, performed in neonatal intensive care units under specific medical indications, that requires a high level of expertise [1]. It should be performed only by trained personnel in a neonatal or paediatric intensive care unit equipped with complete monitoring and with the capacity to perform resuscitation if necessary [2]. The need for exchange transfusion has notably decreased with the prevention of haemolytic disease due to rhesus (Rh) incompatibility and with the systematic application of the clinical practice guidelines of the American Academy of Pediatrics (AAP), published in 2004, for the identification and treatment of new-born babies at risk of severe hyperbilirubinemia [3]. Because exchange transfusion is a rare procedure, paediatricians in training do not have experience with it. Opportunities in daily practice to develop skills in exchange transfusion are rare, and professionals in paediatrics do not reach a sufficient level of competence to master this procedure in the clinical setting [4].

Clinical simulation scenarios and digital teaching environments are postulated as valid and effective teaching options for the development of competencies in medical personnel in training at the postgraduate level [5]. The use of simulation-based training as an educational tool has become an increasingly accepted method for improving the skills of health personnel in a safe learning environment [6]. Simulation standardizes and guarantees exposure to a specific event, allows the teaching of specific clinical skills, decreases the generation of error, increases the safety of the patient and the student, and improves clinical judgement [7–10]. On the other hand, the use of digital environments facilitates learning through interactive tools and is postulated as an effective model for the acquisition of knowledge in medical schools.

We are not aware of reports on the use of simulated learning strategies compared to strategies based on digital didactic environments, particularly in the evaluation of competencies in exchange transfusion.

The implementation of educational strategies related to exchange transfusion during the training of future paediatricians could positively impact the health outcomes of patients who require this procedure [11]. This protocol, through a controlled clinical trial, seeks to compare two educational interventions in exchange transfusion, one based on simulation and another based on the development of a digital didactic environment, for the development of competencies in exchange transfusion performed by postgraduate students in paediatrics.

### State of the art

The use of simulation to teach the knowledge, skills and behaviours necessary for effective paediatric resuscitation has become widespread and has been massively adopted by paediatric institutions; it is of vital importance in topics such as neonatal resuscitation. Studies such as that of Sawyer [12] suggest that simulation education is associated with improved performance in neonatal resuscitation skills and propose the use of simulation in training. Gaies [13], using a randomized controlled trial, compared the standard teaching method with simulation-based modules for training in the performance of frequent paediatric procedures, such as bag mask ventilation, venipuncture, peripherally inserted central catheter and lumbar puncture; it was shown that the participants in the simulation group performed the peripheral insertion of the central catheter more successfully than the controls (78% vs. 35%; *P* = 0.01) and obtained significantly higher scores on the knowledge tests for various procedures and on checklists for the peripheral insertion of a central catheter (81% vs. 61%; *P* = 0.003) and lumbar puncture (77% vs. 68%; *P* = 0.04) in the initial evaluation [13]. Sudikoff studied and evaluated the effectiveness of a simulation session and teamwork in the management of the paediatric airway by paediatric residents in comparison with a group that did not receive the simulation intervention, and found that the intervention group had higher scores (*P* < 0.05), fewer adverse events and more positive teamwork [14].

On the other hand, in relation to teaching in digital didactic environments in paediatrics, Kessler et al. conducted a randomized controlled trial in paediatric residents and proposed that there is no difference in the effects of lumbar puncture audiovisual training combined with simulation versus audiovisual training alone. Despite better success rates in the first simulated attempt immediately after audiovisual training, with control 6–8 months later, no statistically significant differences were found between the groups in their comfort, knowledge base or performance on the Objective Structured Clinical Examination (OSCE) [15]. Tofil et al. conducted a prospective observational cohort study to evaluate the comfort and performance of second-year residents in simulated resuscitation scenarios and procedural skills, particularly intraosseous needle insertion and intubation, as a result of weekly 1-h sessions during paediatric intensive care rotations accompanied by video simulation assistance; their study showed that the intervention increased the residents’ general confidence in the management of various paediatric resuscitation events and their perceived capability [16].

## Methods

### Study design

This is a randomized, parallel, single-blind trial with 1:1 allocation to the interventions studied.

### Study subjects

Students in the paediatrics specialization, who meet the inclusion criteria and who wish to participate voluntarily and sign informed consent, will be included. In Colombia, there are currently 700 students in the paediatrics specialization, who are members of the Colombian Society of Pediatrics, and all of them are going to be invited by e-mail, to participate in the study. The inclusion criteria are as follows:

- Men and women of legal age who are students enrolled in a paediatrics specialization programme at Colombian university medical schools
- Students in any year of specialization in paediatrics

The exclusion criterion is participation in rotation outside Columbia as part of the curriculum.

### Interventions

#### Detailed description of the procedure

The study protocol was developed in the simulation laboratory (simulated hospital) of the Universidad de La Sabana (University of La Sabana), and in a virtual didactic environment. Planning and structuring will be carried out by neonatology and paediatric medical education teachers at the School of Medicine of the Universidad de La Sabana (University of La Sabana).

**Fig. 1:**
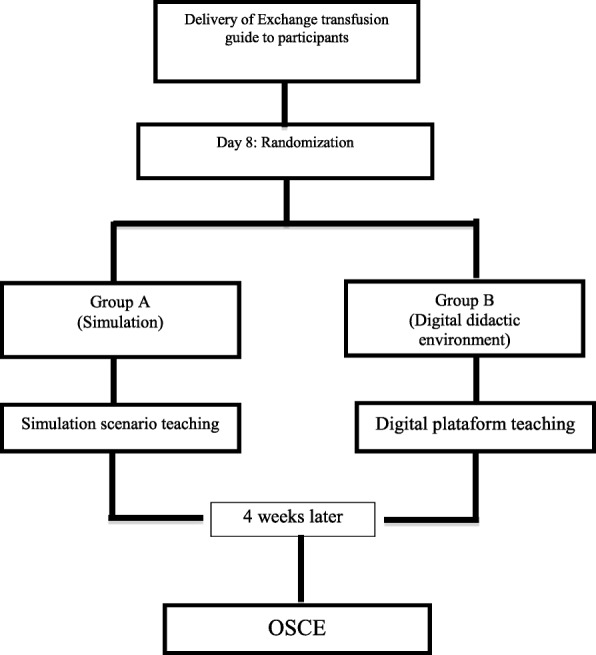
Flow diagram of enrolment, intervention, and assessment. OSCE, Objective Structured Clinical Examination

Once a student has agreed to participate and provided signed informed consent, he or she will be entered into the study protocol (Fig. 1). Initially, students all participating students will receive a theoretical guide on exchange transfusion to study and analyse on their own in advance of the intervention. This guide is the only document that will be provided, prior to randomization. The guide includes aspects such as indications for exchange transfusion, objectives, step-by-step technique, preparation of the newborn, necessary supplies for implementation and complications.

**Fig. 2:**
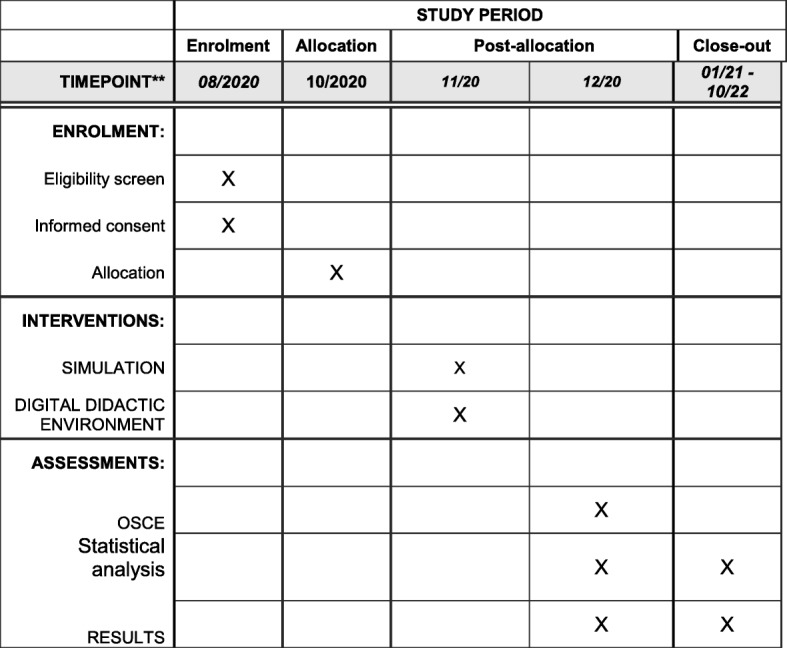
Schedule protocol. OSCE, Objective Structured Clinical Examination

Subsequently, the students will be assigned to an educational intervention (simulated scenario or digital didactic environment) by randomization in permuted blocks (see “Randomization and blinding”) (Fig. 2). Both educational techniques will have the following objective: the student will be able to identify, know and/or perform the indications of the procedure, the preparation and disposition of the supplies and materials necessary for the procedure, the preparation of the newborn for exchange transfusion and the step-by-step procedure. The two techniques will be implemented as follows:

- Simulated scenario:- The educational intervention will be developed in the simulation laboratory. - The students will observe the exchange transfusion procedure and will be able to practice in the procedure under the supervision of a neonatology expert. - The neonatologist in charge of the training of the simulation group, knows from the beginning the assigned intervention group. - For the training, neonatal high-fidelity simulators, clinical history and paraclinical exams, necessary supplies for the procedure, a simulated mother, a professional nurse and a nursing assistant will be available. - Training will take place in individual skill stations (identification of indications, communication, management of medical devices and procedures) and in integrated clinical scenarios.
- Digital didactic environment:- Students assigned to this group will receive access to a digital didactic environment where they will have at their disposal the exchange transfusion guide and a complete video explaining the indications, the preparation of supplies and the newborn for the procedure, the management of medical devices required and a step-by-step description of the exchange transfusion technique. - The student will see an integrated clinical case. - This video will be developed by the neonatology and paediatric medical education teaching team in the simulation laboratory of the university using neonatal high-fidelity simulators, a simulated mother and the necessary medical equipment and supplies (the video can only be seen by each participant, a maximum of three times).

At 4 weeks, after the training is over, the students will be scheduled for a practical evaluation of the procedure in the simulation laboratory at the Universidad de La Sabana (University of La Sabana) (Fig. 2). For this evaluation, a simulated scenario will be developed using a standardized clinical case with a simulated patient according to the OSCE methodology. All participation sessions during the OSCE evaluation will be video and audio recorded to allow the reviewer/evaluator, who will be blinded to the educational intervention assigned to the student, to apply the checklists at a later time. In order to maintain the participant students, we will use study through materials and e-mails, during the time period of the study.

#### Outcome measures

The outcome measures are as follows:

- Main outcome: overall result of the OSCE evaluation (checklist). The performance score will be the score obtained for the evaluation divided by the maximum possible score of the test and multiplied by 100. A superior performance is defined as a score greater than or equal to 85%, and a non-superior performance is defined as a score equal to or less than 84%.
- Secondary outcomes: performance on the OSCE checklist in the following sub-areas:
- Clinical history and approach to patient risk and indication for exchange transfusion.
- Comprehensive assessment of the patient with neonatal jaundice.
- Management of the patient with neonatal jaundice and indication for exchange transfusion.

The OSCE and the instrument (checklist) for evaluating students were developed using consensus methodology performed by a panel of neonatologists or paediatricians who are experts in the exchange transfusion procedure. Subsequently, in a second phase, the validity and reliability of the OSCE created by consensus will be tested.

The instrument consists of the following areas, within which the items to be evaluated are described:

- Clinical history of and assessment of risk to the patient with neonatal jaundice: The student should obtain information about the risk factors to classify patients through an interview with the parents and the study of the clinical history.
- Comprehensive assessment of the patient with neonatal jaundice: the student should evaluate the patient’s need for exchange transfusion by performing a physical exam and requesting paraclinical tests and their interpretation. The student should indicate the treatment and communicate the decision to the parents.
- Management of the patient with neonatal jaundice and indication for exchange transfusion: the student must perform exchange transfusion in the newborn and, to do so, must prepare the necessary elements, determine the replacement fluid and volume, evaluate the patient’s condition, prepare and monitor the newborn and identify possible complications.

Each item will be classified on an ordinal scale: 2 points if the student fulfils the entire evaluated item correctly, 1 point if the student partially meets the evaluated item, and 0 if he or she does not. The total score obtained will be divided by the maximum possible score and multiplied by 100.

### Sample size

No previous studies were identified that compared simulation to a digital platform in the development of exchange transfusion competencies. A randomized trial that compared success rates in the performance of frequent paediatric procedures, such as bag mask ventilation, venipuncture, peripherally inserted central catheter and lumbar puncture, identified advantages with simulation compared to standard teaching. This study identifies absolute differences in the likelihood of success of 33%, 20% and 9% in favour of the simulation for the insertion of a central line, peripheral catheter and lumbar puncture, respectively [13]. A 30% difference in the probability of having an acceptable exchange transfusion procedure is considered acceptable when comparing the two teaching interventions [13]. Based on this difference, and for power of 80% and a type I error level of 5%, 80 participants are required (40 per group).

### Randomization and blinding

Students will be assigned to the study groups using a computer-generated randomization sequence. This process will be centralized in the paediatric office of the Universidad de La Sabana (University of La Sabana). The sequence will be generated through the Research Randomizer page (www.randomizer.org), and a permuted blocks strategy with a block size of 8 will be used. The permuted blocks strategy and the size of the blocks will not be known by the researcher. The random sequence be concealed by placing the allocation in opaque envelopes at the time of assignment.

The researcher and the student cannot be masked to the allocation due to the nature of the interventions. However, the research team responsible for data analysis will be blinded to the allocation as will the expert neonatologist who evaluates the video-recorded procedures using the checklist. The database will be coded and the identification code will not be available to the analyst.

### Statistical analysis

The participants will be described using descriptive statistics. Quantitative variables will be presented as measures of central tendency and dispersion, and qualitative variables as absolute and relative frequencies.

Effect of the intervention: the outcome of the study will be a score equal to or greater than 85% on the OSCE; therefore, the outcome variable will be dichotomous. The chi-square independence test (or the Fisher exact test if the assumption of at least 5 counts per cell is violated) will be performed to evaluate the effect of the interventions. Additionally, relative risk and the respective 95% confidence interval will be estimated as a measure of effect.

Multivariate analysis will be performed using a non-conditional logistic regression model, with the outcome of the study as the dependent variable and the intervention and year of training as the independent variables. Interim analyses will not be performed. Two-tailed analyses will be performed for a type I error level of 5%. The Stata 15® programme will be used in the analyses.

## Discussion

Exchange transfusion is a procedure that requires expertise to achieve adequate patient outcomes. Currently, the number of neonates that require this procedure is decreasing due to prevention efforts and the adoption of management guidelines worldwide; however, it continues to be a procedure that every paediatrician should perform adequately and for which, given the current low level of exposure in the hospital environment, it is not possible to achieve an adequate training curve. The inclusion of new educational strategies, such as simulation and digital educational environments, is being considered as an option for increasing exposure.

Simulated practice is an experimental learning tool that offers didactic innovation, motivation for learning about health and improved learning opportunities; it allows the acquisition of skills, the improvement of knowledge and preparation for emergency situations [13]. It offers a useful method for acquiring procedural skills that are important in daily practice, providing an educational bridge to prepare students for reality and allowing the paediatrician to strengthen his or her knowledge, thus reducing patient morbidity and mortality [17].

Digital didactic environments provide opportunities for self-directed learning, interaction and substantial periodic changes in communication models and knowledge production processes. Only with the adequate use of technological media in the service of education and the construction of knowledge will we avoid risks and allow the full personal and social development of students, and the creation and recreation of the teaching and learning processes and of thought and intellectual work [18].

There are no reports on the use of simulated strategies or digital didactic environments for training on exchange transfusion. This indicates the need to develop such training methods which, although they cannot replace real clinical scenarios or direct learning with patients, are complementary in teaching-learning in health sciences and can be integrated into curricular developments [19, 20].

The potential limitations of this protocol are as follows. External validity: this study will be performed only by Colombian paediatrics residents, therefore, the results will only be applicable this group. Follow-up time frame: the evaluation will be performed after 4 weeks of the intervention, and the maintenance of competencies over time will not be verified, afterwards. Participant dropouts: even though this could be a limitation, we expect these to be below 10%.

## Trial status

The clinical trial has not yet initiated the recruitment of participants.

Protocol Amendment Number: 03. Issue Date: 9 March 2020.

Date recruitment began: November 2020.

Date when recruitment will be completed: June 2022.

## Data Availability

The datasets used and/or analysed during the current study are available from the corresponding author on reasonable request.

## Abbreviations

OSCE: Objective Structured Clinical Examination
APP: American Academy of Pediatrics
